# Transcending Borders and Bias: The Multifaceted Life and Legacy of Dr. Hector Pérez García

**DOI:** 10.7759/cureus.45232

**Published:** 2023-09-14

**Authors:** Muhammad Hamza Shah, Matthew D Turner

**Affiliations:** 1 Medical School, Centre for Anatomy, Deanery of Biomedical Sciences, The University of Edinburgh, Edinburgh, GBR; 2 Medical School, School of Medicine, Dentistry and Biomedical Sciences, Queen's University Belfast, Belfast, GBR; 3 Emergency Medicine, Penn State Health Milton S. Hershey Medical Center, Hershey, USA

**Keywords:** activism, united states, military physician, hispanic immigrants, hector perez garcia

## Abstract

Héctor Pérez García's transformative leadership in Hispanic civil rights within the U.S. remains an integral topic of academic discussion. As a dedicated physician, a resilient World War II veteran, and a fervent civil rights advocate, García seamlessly merged these roles, paving a distinct path that defined his multifaceted advocacy. His unique approach and steadfast commitment to justice and equality not only solidified his position as a transformative leader but also emphasized the importance of his endeavors in shaping the nation's historical narrative. It is this intricate interplay of his personal experiences and professional pursuits that places García at the epicenter of academic discussions around Hispanic rights and activism. This paper is committed to unpacking the nuances of García's significant contributions, while also providing a comprehensive perspective on the socio-political landscape of his time - a setting that both shaped and was profoundly affected by his groundbreaking efforts

## Introduction and background

Hector P. García's birth on January 17, 1914, in Llera, Tamaulipas, coincided with a tumultuous epoch marked by revolutionary fervor in Mexico. The sweeping social and political unrest during this era inevitably influenced the trajectory of his life. As Mexico grappled with internal strife and profound ideological transitions, the García family, cognizant of the perils of nurturing a family amidst such chaos, sought sanctuary in the United States when Hector was merely four [1]. Hailing from Mexico's emergent middle class, Hector's parents - José and Faustina García - were entrenched in the realm of education. While José maintained a politically neutral disposition, Cleotilde, Hector's sister, would later recall his initial proclivity towards the ideals of the Mexican Revolution. This inclination was, in part, a response to his consternation about the overpowering influence the Catholic Church exerted on Mexican society, an influence that remarkably persisted even after the restrictions post the War of Reform.

The catalyst for the García family's migration to the U.S. was twofold: the omnipresent threat of revolution-induced violence and the deleterious effects of a drought on their modest ranch. On reaching American soil, they established themselves in Mercedes, Texas, where their lineage - tracing back to Spanish Jews escaping the Catholic Inquisition in search of sanctuary in Mexico - had previously settled [2]. Over successive generations, these ancestors gradually concealed their Jewish heritage, embracing Catholicism instead. Upon arrival in the U.S., José procured employment with the American Rio Grande Land & Irrigation Company situated in Thayer, Texas, before the family's eventual relocation to Mercedes. It was in this milieu, shadowed by the remnants of the Black Jack Pershing Expedition and apprehensions of the Mexican Revolution's encroachments, that young Hector cultivated a profound respect for the military, a sentiment he cherished throughout his lifetime.

In their quest for stability and prosperity in the Rio Grande Valley, the García family encountered numerous obstacles. Despite José not achieving his personal aspirations in Texas, dedicated himself to ensuring his children's success, instilling in them values of discipline, hard work, and resilience. This holistic upbringing, encompassing home, school, work, and civic participation, later shaped Dr. García’s advocacy for the comprehensive development of Mexican Americans. Eventually, through sheer resilience, José and Faustina succeeded in raising a brood of accomplished individuals: José Antonio, Hector, Emilia, and Cleotilde, each leaving an indelible mark on the García family's legacy.

## Review

Education

Dr. García's educational journey began at Edinburg Junior College, located about thirty miles north of his family's residence in Mercedes. The practical decision for him to attend Edinburg was due to his family's financial constraints, even though he had the acumen to attend a more prominent institution. Interestingly, his college commute, often undertaken by hitchhiking, epitomized his dedication and García family's belief in the value of education [2]. His father's reminder that this was "the price of an education" exemplifies the family's ethos. 

Dr. García’s time at Edinburg Junior College was transformative. His distance from his father's overbearing influence allowed him to mature, feeling more independent. At Edinburg, he connected with a few other Mexican American students, sharing a bond of navigating a challenging environment that wasn't always welcoming to their community. His positive outlook on education, combined with his tenacity, allowed him to excel at the institution. Similarly, when he transitioned to the University of Texas, he found it to be a place of profound learning and opportunity. Here, he felt privileged, not only for the vast resources available but also for the chance to engage with the state's elite. The university was a world apart from his experiences at Edinburg, with its grandeur and promise laying the foundation for what was to come in his life [2]. However, as a pre-med student, his primary focus remained steadfastly on his academic pursuits. As such, he often seemed to be on the periphery of the reform conversations, not diving deep into the intricacies of Mexican American history, even though he was surrounded by figures deeply engaged in these subjects. Dr. García’s memories of the university suggest minimal racial tensions, an experience that appears contrary to what other Mexican American students felt. Like Mike De La Fuente, a talented baseball player, some Mexican Americans had to continuously assert their identity and face discrimination head-on [3]. By 1936, he graduated with a bachelor's degree in zoology but couldn't attend his graduation due to lack of funds. This highlighted the financial struggle he and many like him faced at the time. Eventually choosing to study medicine, he followed in the footsteps of his brother Antonio at the University of Texas Medical School in Galveston. While Dr. García was known for his assertiveness later in life, during his studies, he chose to adapt and overcome obstacles rather than directly challenge them. This tactic allowed him to focus on his education and laid the foundation for his future skills.

At medical school, he also found his Spanish background helpful in understanding Latin terms. Socially, Galveston was more isolating than Austin for García, but he managed to connect with a few individuals. García's time in Galveston was marked by his proactive involvement in the local community, particularly focusing on disease prevention for Latino communities. He, along with volunteers, taught about hygiene, immunization, and various diseases. García’s dedication to these communities won him admiration and deep respect. His initiatives included translating health literature from English to Spanish, made possible with the help of a generous Italian family in Houston. Engaging with the local community [4]. He was frequently invited to local events and celebrations, making him a vital part of the active community. This engagement not only provided him with social opportunities, but also taught him about organizing within different classes of the Mexican community.

Soon after García graduated, he was faced with yet another struggle. In the 1940s, prejudice against Hispanic doctors was rampant, especially in southern states. While it was one thing to allow a minority student to earn a degree, granting them the responsibility and recognition of a residency was another matter entirely. This implied trust, and unfortunately, racial prejudices often interfered with the judgment of those in charge. Yet, it was this very prejudice that led him to Omaha, a turn in his journey he had never anticipated. Omaha in the 1940s was also not devoid of racial prejudices. However, St. Joseph’s Hospital stood as an exception. The hospital's willingness to recruit outside of the conventional pool of candidates was not just a testament to their progressive views, but also a practical strategy. They recognized the potential and talent that others overlooked because of blind prejudice [2, 4]. For Dr. García, this was a golden opportunity, a chance to prove his mettle and break the stereotypes that held back many of his peers.

Military career

Dr. García's commitment to military service and his nation began early in his life. At the tender age of fifteen, he enrolled in the Citizens Military Training Corps in 1929, reflecting his nascent passion for military service. This rigorous training would not only equip young Héctor with the foundational knowledge required for serving in the U.S. Army but also refine his interpersonal skills and familiarize him with the hierarchical chain of command. His exemplary discipline and commitment quickly earned him recognition. By June 8, 1935, Dr. García was commissioned as an infantry officer, bearing the title of Lieutenant García [5]. Notably, within a couple of months, on August 12, 1935, he received commendation as a second lieutenant with the 357th Infantry at Camp Bullis, Texas [2]. This honor acknowledged his swift and effective actions during a storm, particularly in restoring interrupted utilities. At just 21, such recognition was not only a testament to his skill but also a source of immense pride for him.

However, for someone as driven as Dr. García, one commendation was merely a stepping stone. Four years later, on August 12, 1939, he received another commendation. Major C.C. Patterson praised both Lt. Héctor P. García and Lt. Theo A. Bowie for their exceptional work during an active training period [2]. Serving respectively as Communications Officer and Transportation Officer, both officers were lauded for their efficiency and commitment to their roles. Upon completing medical school at the University of Texas Medical Branch-Galveston in 1940, Dr. García’s rank was elevated to first lieutenant. With an unwavering focus on growth, by December 16, 1941, he was inquiring about a promotion to captain of the infantry. His dedication to serve was palpable in his letters, as he expressed his eagerness to join the Medical Corps to aid the army's acute need for doctors. Moreover, he was willing to undertake foreign duty if required. Correspondences between First Lieutenant García and the Headquarters First Military Area suggest a back-and-forth regarding his transfer from the infantry to the Medical Corps. However, by August 15, 1942, his determination paid off when he finally transitioned to the Medical Corps, with a promotion to captain following a month later [2, 4].

**Figure 1:**
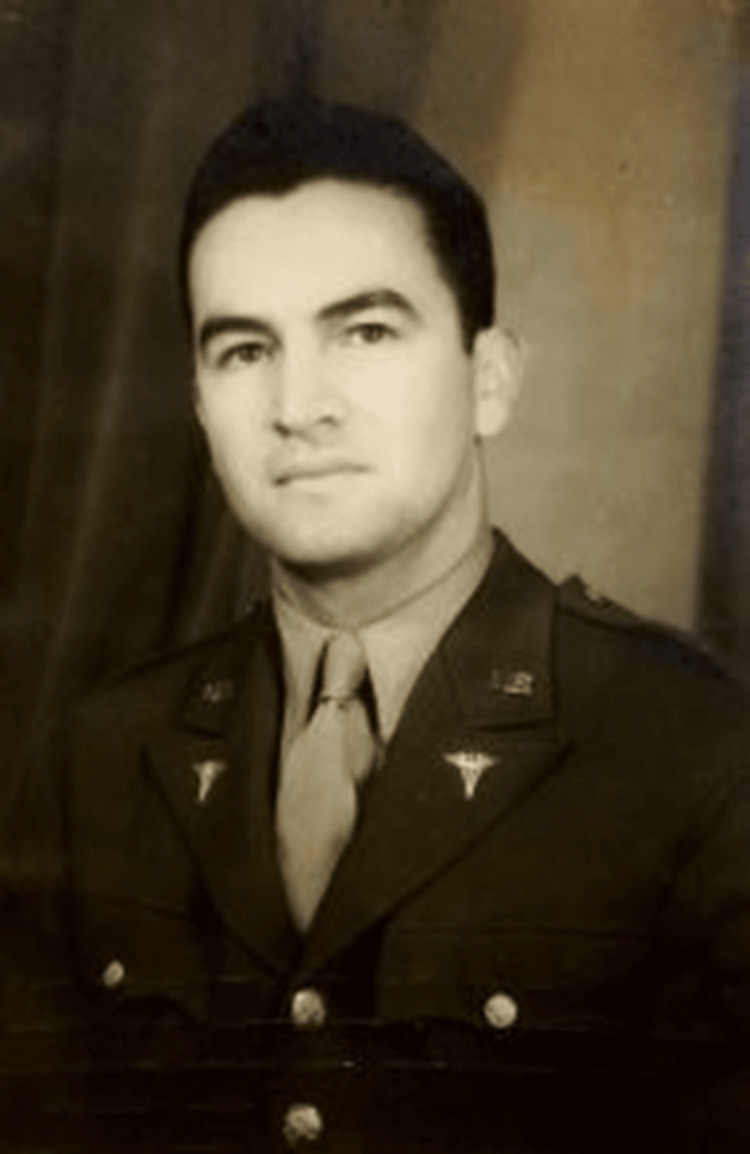
Major Héctor P. García pictured in military uniform while stationed in Tunisia in 1944. Source: "Major Héctor P. García in uniform, Tunisia, 1944", featured in "The Inspiring Life of Texan Héctor P. García" by Lisa Taylor (available at https://blogs.loc.gov/folklife/2016/09/the-inspiring-life-of-texan-hctor-p-garca/)

García’s service spanned several notable theaters of World War II, including Tunisia, Italy, and central Europe (Figure 1). His meritorious service was recognized with the Bronze Star Medal in 1945 for outstanding diligence and loyalty. Furthermore, his record reveals a range of awards such as the European-African-Middle Eastern Campaign Medal along with six Battle Stars [6]. Amidst his professional accomplishments, there were personal anecdotes that painted a vivid picture of the times. A memorable interaction with General Patton in Italy highlighted biases and misconceptions prevalent in those days. Remarkably, despite his significant service to the nation, García only received his U.S. citizenship in 1946. 

American GI Forum

Upon returning to Texas post-World War II in 1946, Dr. García was met with a combination of relief, anticipation, and a burning desire for change. Though he had witnessed the harrowing realities of war, including the death and poverty that came with it, he was soon confronted with another challenge: the shattered promises made to his fellow veterans. Feeling an intrinsic need to do more, he became involved with the League of United Latin American Citizens (LULAC) [7]. Established in Corpus Christi in 1929, LULAC's mission centered around combating ethnic discrimination in the U.S., focusing on poverty, healthcare, and education. While he served as its local president in 1947, he felt a void, particularly when it came to addressing the distinct needs of the veterans.

**Figure 2:**
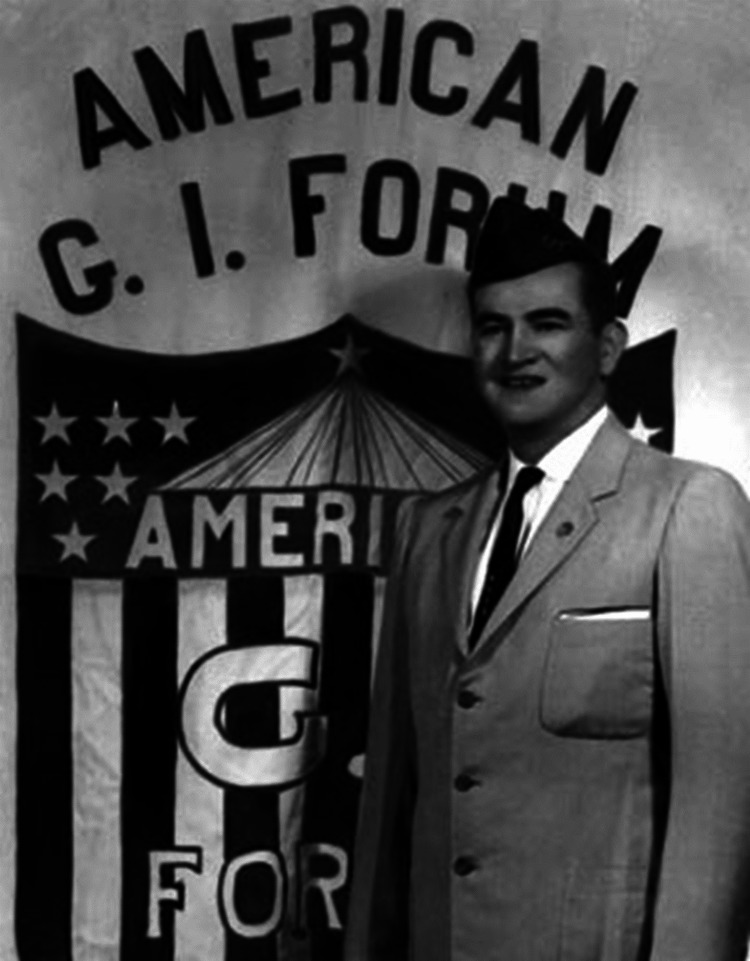
Dr. García in the 50s at an American GI Forum event. Source: "Dr. Héctor P. García standing in front of the American GI Forum banner, early 1950s" by American GI Forum, licensed under Creative Commons CC0 License (available at https://commons.wikimedia.org/wiki/File:Hector_P._Garcia.jpg)

It was this spark that led to a landmark event in March 1948. A call was sent out to all veterans, inviting them for a pivotal gathering at Lamar Elementary School in Corpus Christi. The overwhelming response saw seven hundred veterans convene, leading to the historic formation of the American GI Forum on March 26, 1948, with Dr. García as its founder and leader until 1996 (Figure 2). This organization not only addressed veterans' issues but also broader civil rights concerns for the Hispanic community. One of the Forum's defining moments was the Felix Longoria Incident, which resulted in a national outcry over the prejudice faced by Mexican-American veterans. The incident catalyzed the expansion of G.I. Forum chapters to other states.

However, beyond the organization's expansion, the Forum would soon become the touchstone of a crucial civil rights movement. The case of Felix Longoria in 1948 was a turning point. Mrs. Beatrice Longoria's tragic quest to give her husband, a decorated World War II hero, a decent funeral brought her to Dr. García [8]. Denied a wake service at Rice Funeral Home because of his ethnicity, Felix’s story became a watershed moment for the American GI Forum. Their plight caught the attention of then-senator Lyndon B. Johnson, leading to Felix's burial with full military honors at Arlington National Cemetery.

This event propelled Dr. García and the American GI Forum into the national spotlight, intertwining veterans' rights with broader civil rights issues. Their advocacy bore fruit with significant cases like Hernandez v. Texas in 1954, which addressed the systematic exclusion of Mexican-Americans from juries, and Brown v. Board of Education, which declared racial segregation in public schools unconstitutional. The Forum also played a pivotal role in the Cisneros v. Corpus Christi Independent School District case [9], which identified the intentional segregation of Mexican American and black students.

Advocacy and activism

In an unexpected twist, President Lyndon Johnson expressed his desire to appoint Dr. García as an ambassador to the United Nations [2]. This prestigious role would require him to relocate to New York City, a world away from Corpus Christi, his family, and his medical practice. Upon the potential appointment, the Federal Bureau of Investigation (FBI) agents descended upon Corpus Christi, scrutinizing Dr. García's life, contacts, and relationships.

On September 20, 1967, amidst a blend of excitement and skepticism from various quarters, Dr. García received his commission to the United Nations from Ambassador Arthur Goldberg [10]. The very next month, he was accorded the personal rank of Ambassador, a remarkable achievement not just for him, but also for the broader Mexican American community. The Roosevelt Hotel in New York City became his new residence, a far cry from his life in Corpus Christi. The urban allure of New York, combined with the responsibilities and privileges of his role, transformed his daily existence. Yet, Dr. García's core remained unchanged. His affable nature ensured that he was both liked and respected at the United Nations. In addition, Dr. García chose to address the General Assembly in Spanish, emphasizing the significance of relations with Latin America. Delivered on October 26, 1967 [11], this speech not only underscored his fluency in Spanish but also challenged prevailing stereotypes. However, as 1967 neared its close, a longing for Corpus Christi and his family began to overshadow the allure of diplomatic life [4]. By December, Dr. García made the difficult decision to resign from his post, returning to the embrace of his medical practice, family, and advocacy work in Texas. His stint at the United Nations had broadened his horizons and further cemented his reputation as a statesman and advocate.

Dr. García's advocacy also extended to political campaigns and collaborations with U.S. presidents. He was particularly instrumental in mobilizing the Hispanic vote for John F. Kennedy in 1960. For his relentless service, he was awarded the Medal of Freedom by President Ronald Reagan and had a savings bond bearing his portrait issued by the U.S. Treasury [12]. 

Legacy

Dr. García's vision was simple: all Americans deserve equal freedom and opportunity. Consequently, Dr. García's receipt of this esteemed medal in 1984 not only underscored his remarkable accomplishments but also highlighted the evolving landscape of American recognition. As the first Mexican American to receive this award, Dr. García's honor broke barriers and served as a testament to his lifetime of service. From advocating for healthcare, veterans’ rights, civil rights, to education, his contributions echoed the spirit of the medal: a lifetime dedicated to bettering the nation.

## Conclusions

From the tumultuous backdrop of revolutionary Mexico to the racially charged soils of Texas, Dr. García’s story is one of relentless resilience. Born to a family fleeing the clutches of the Inquisition and shaped by migration's ebb and flow, Dr. García's life turned out to be a dance between adversity and aspiration. Whether hitchhiking to education, fighting in WWII, or rallying for the rights of his community with the American GI Forum, he remained a beacon for change. All in all, his journey epitomizes the power of persistence, reminding us that one man's voice can indeed echo through the annals of history.
